# Mechanism of Collaborative Enhancement of Binding of Paired Antibodies to Distinct Epitopes of Platelet Endothelial Cell Adhesion Molecule-1

**DOI:** 10.1371/journal.pone.0169537

**Published:** 2017-01-13

**Authors:** Raisa Kiseleva, Colin F. Greineder, Carlos H. Villa, Elizabeth D. Hood, Vladimir V. Shuvaev, Jing Sun, Ann-Marie Chacko, Valsamma Abraham, Horace M. DeLisser, Vladimir R. Muzykantov

**Affiliations:** 1 Department of Pharmacology, The Perelman School of Medicine, University of Pennsylvania, Philadelphia, PA, United States of America; 2 Pulmonary, Allergy & Critical Care Division, The Perelman School of Medicine, University of Pennsylvania, Philadelphia, PA, United States of America; 3 Laboratory for Translational and Molecular Imaging, Duke-NUS Medical School, Singapore, Republic of Singapore; 4 Corporal Michael J. Crescenz Veterans Affairs Medical Center in Philadelphia, PA, United States of America; New York State Department of Health, UNITED STATES

## Abstract

Monoclonal antibodies (mAbs) directed to extracellular epitopes of human and mouse Platelet Endothelial Cell Adhesion Molecule-1 (CD31 or PECAM-1) stimulate binding of other mAbs to distinct adjacent PECAM-1 epitopes. This effect, dubbed Collaborative Enhancement of Paired Affinity Ligands, or CEPAL, has been shown to enhance delivery of mAb-targeted drugs and nanoparticles to the vascular endothelium. Here we report new insights into the mechanism underlying this effect, which demonstrates equivalent amplitude in the following models: i) cells expressing a full length PECAM-1 and mutant form of PECAM-1 unable to form homodimers; ii) isolated fractions of cellular membranes; and, iii) immobilized recombinant PECAM-1. These results indicate that CEPAL is mediated not by interference in cellular functions or homophilic PECAM-1 interactions, but rather by conformational changes within the cell adhesion molecule induced by ligand binding. This mechanism, mediated by exposure of partially occult epitopes, is likely to occur in molecules other than PECAM-1 and may represent a generalizable phenomenon with valuable practical applications.

## Introduction

Targeting drugs to endothelial cells has the potential to improve treatment for diseases involving inflammation[1,2], thrombosis[3], ischemia[4], and tumor growth[5,6]. Endothelial delivery is typically achieved by conjugation of drugs or carriers to affinity ligands, such as monoclonal antibodies (mAbs) or their fragments, which bind to endothelial surface determinants. Successful translation of these strategies to the clinical domain mandates thorough investigation of the mechanisms by which affinity ligands interact with their target molecule, as well as the functional consequences of this interaction.

Numerous animal and cell culture studies have identified PECAM-1 (CD31) as an attractive target molecule for endothelial drug delivery [1,7–9]. A 130-kDa member of the immunoglobulin (Ig) gene superfamily, PECAM-1 consists of six extracellular, Ig-like domains, a transmembrane domain of 19 residues, and an 118 amino acid cytoplasmic tail [10]. It is expressed on the surface of vascular endothelium and hematopoietic cells (platelets and leukocytes) at high and modest levels, respectively [11].

Homophilic contacts between PECAM-1 molecules are involved in many endothelial functions [10]. Endothelial PECAM-1, which localizes predominantly in intercellular junctions, mediates the extravasation of leukocytes at sites of inflammation [12], serves as a flow sensor, transduces signaling and maintains the vascular integrity of endothelial monolayer [10]. X-ray crystallography revealed that the homophilic binding region is located within the first two extracellular domains of PECAM-1 monomers that form interfaces between adjacent IgD1/D1, IgD1/D2 and IgD2/D2 domains [10].

Anti-PECAM-1 monoclonal antibodies (mAbs) serve as affinity ligands for designing new drug delivery systems [13]^,^[14]^,^[15]. It has been fortuitously discovered that binding of mAbs to PECAM-1 enhances binding of other ("paired") mAbs to adjacent distinct epitopes [11]. This phenomenon, to which we have given the moniker “Collaborative Enhancement of Paired Affinity Ligand", or CEPAL, improves endothelial targeting of *in vivo* as enhanced pulmonary uptake of radiolabeled PECAM-1 mAb was observed after intravenous co-injection with unlabeled paired mAb.

Multifaceted functionality of PECAM-1 molecule, involved in vascular integrity [16], hematopoiesis [17], inflammation and angiogenesis [18] necessitates understanding of mechanism and consequences of CEPAL. A systematic investigation of the mechanistic and drug delivery implications of this enigmatic phenomenon is warranted. Described herein are the efforts focused on investigating the role of cellular activation and PECAM-1 interactions in the mechanism of CEPAL.

## Materials and Methods

### Cell lines

Human malignant mesothelioma cells (REN) [19] stably expressing recombinant mouse PECAM-1 (RmP) were maintained in RPMI-Glutamax supplemented with 10% (v/v) FBS, 1% (v/v) A/A, and 250 μg /ml G418. Wild type REN cells (REN-WT) were used as a control. REN cells expressing mutant PECAM-1 (RmP_K89A_) were cultured in RPMI complete media with 1 μg/ml puromycin.

### Antibodies and other reagents

Hybridoma for anti-mouse PECAM-1 monoclonal antibody 390 (rat IgG2a) was originally generated in the rat by immunization with a mouse 32D leukocyte cell line and screened against muPECAM-1Δ12,15 and was a generous donation of Dr.Steven Albelda (University of Pennsylvania, Philadelphia, PA) [20], and Mec13.3 (rat IgG2_a_) was purchased from BioLegend (San Diego, CA). Anti-pan Cadherin antibody [CH-19] was purchased from Abcam (Cambridge, MA) (Western Blotting 1:1000). Recombinant Mouse CD31/PECAM-1 Protein, CF was purchased from R&D Systems Inc. (Minneapolis, MN).

### Cellular homogenates

Cells were grown to 90% confluency in 15-cm dishes, washed with phosphate-buffered saline, dislodged using ice-cold Buffer A (20mM Tris, 2mM EDTA, Complete protease inhibitor, pH 7), and scraped off into 50 ml conical tubes. This solution was homogenized for 15 s with an ultrasonic homogenizer (Fisher Scientific, PA) on ice and pelleted by centrifugation (2000 x g, 5 min at 4°C) to remove unbroken cells and larger debris. The supernatant was then centrifuged at 34,000 g at 4°C for 60 min (Beckman Optima XL-100 K Ultracentrifuge, Beckman Coulter, CA). The supernatant was then reconstituted in Buffer A. Membrane protein was quantified using the copper bicinchoninic acid method (Pierce, Rockford IL), with bovine serum albumin (BSA) used as the standard. The PECAM-1 presence in cellular homogenates was confirmed by protein western blot. Cell membranes were stored at −80°C before use.

### Construction of REN cells expressing extracellular domain mutants of muPECAM-1 (RmP_K89A_ cells)

#### Generation of a lentiviral vector expressing the full length murine PECAM-1 cDNA and the mutant

In order to express full length murine WT pecam-1 and its mutants we used a pCDH lentiviral vector as a backbone. Briefly, PECAM-1 cDNA were PCR amplified from pcDNA3 pecam-1 vector.

The sequences of the primer pair used to generate the full length pecam-1 were: 5’-AGATTCTAGA*GCTAGC*ATGCTCCTGGCTCTGGGACTC-3’ (pCDH Pecam-1 FL forward primer) 5’-CAGATCCTT*GCGGCCGC*TTAAGTTCCATTAAGGGAGCCTTC-3’ (pCDH pecam-1 reverse primer), with Nhe1 and Not 1 sequences in italics. In addition to the gene specific sequences, the sequences of the primer pair forward and reverse contained about 16 bp extensions (from both 5’ and 3’ ends) that are homologues to the ends of the destination vector. The lentiviral cDNA expression vector, pCDH-CMV-MCS-EF1- GFP Puro cDNA was obtained from System Biosciences, Mountain View, CA. The PCR amplified full length PECAM-1 was cloned Nhe1 and Not I site of the PCDH lentiviral vector using an In-Fusions Advantage PCR Cloning Kit (Clontech). The DNA sequences of the construct was confirmed by sequencing. The vector was designated as pCDH WT PECAM-1.

### Construction of lentiviral vector encoding mutation in extracellular domain1 mutant of mouse PECAM-1

A mouse PECAM-1 cDNA encoding a mutation in lysine at position 89 to alanine was cloned into the lentviral cDNA expression vector, pCDH-CMV-MCS-EF1-GFP. The mutant K89 was constructed using wild type pcDNA3 PECAM-1 cDNA as the template using Quik Change Lightning Mutagenesis Kit as per manufacturer’s instructions. The sequence of the respective K89 forward and reverse primer pair used to generate the mutant were: 5'-GCACAGTGATGCTGAACAACGCGGAAAAAACCACGATTGAG-3' and 5'-CTCAATCGTGGTTTTTTCCGCGTTGTTCAGCATCACTGTGC-3'. The mutated residue is shown in bold.

### Vector production and concentration

293TN cells were seeded in 10 cm tissue dishes 24 h prior to transfection (3 × 106 cells/dish). Cell culture media was replaced with 9 ml of fresh media containing no antibiotics 2 h before transfection.

Transfection was carried out according to the manufacturer's instructions (System Bioscience). In brief, 293TN cells were co-transfected with 20μl pPACK H1 packaging plasmid mix and 2μg of lentiviral pCDH PECAM-1 vector per each 10 cm dish using pure Fection as a transfection reagent. Twenty-four hours after initiating transfection, the plasmid–pureFection solution was removed, and replaced with complete medium. Cells were cultured for another 24-48h. Lentivirus-containing supernatants was collected at 48 and 72 h after transfection and centrifuged at 3000 x g for 15 min at room temperature (RT) to pellet cell debris. The viral particles are concentrated with PEG-it virus precipitation solution. The viral pellet was resuspended in sterile PBS at 1/100 of the original volume. The viral stock was aliquot in cryogenic vials and stored at −80°C.

Viral titer

After transfection, the viral titer was determined by counting GFP-positive cells by fluorescence microscopy. 293T cells were plated at 5×10^4^ cells/well in a 24 well plate in 1ml Dulbecco’s modified Eagle’s media containing 10% serum, L-glutamine, and antibiotics. Twenty-four hours later, cells in each well were transduced with 5-fold dilutions of vector encoding GFP. Forty-eight hours post transduction, cells were analyzed for GFP expression using fluorescence-activated cell sorting (FACS) (BD FACS Aria II SORP, BD Biosciences, San Jose, CA). Transducing units per milliliter was calculated as follows: # of GFP positive colonies counted × dilution factor × 40.

Transduction of REN cells

One day prior to transduction REN mesothelioma cells were plated in 24-well plates at 5X10 ^4^ cells. After 24 hours, REN cells were infected with lentiviral particles containing full length murine PECAM-1 cDNA or variant of PECAM-1. After 72 h the cells were selected with puromycin (starting concentration 1.5 μg/ml with subsequent lowering the dose to 1.0 μg/ml) in order to establish stably transfected RmP cells. After 14 days the cells were stained with murine PECAM-1 Ab (clone 390) and the cells expressing murine PECAM-1 were sorted using FACS (S1 Fig). The expanded cells were used for further experiments.

### Radiolabeling of antiPECAM mAbs

MAbs were directly radioiodinated using [^125^I]NaI (Perkin Elmer, Waltham, MA) and pre-coated Iodination tubes (Thermofisher, Waltham, MA), and purified over a 2-mL desalting column (Thermofisher, Waltham, MA). The radiolabeling efficiencies were 65–95%, and the radiochemical purity, post-purification, was >95% as assessed by the trichloroacetic acid assay. Protein concentrations were determined by NanoDrop3000 spectrophotometer (Thermofisher, Waltham, MA) and the specific activities of [^125^I]-mAb were calculated to be 5–10 μCi/μg.

### Live-cell PECAM-1-binding assays

Cells were seeded at 40,000 cells/well density (counted by a Z Series Coulter Counter, Beckman Coulter Life Sciences) in 96-strip-well plates (Corning Life Sciences, Lowell, MA). Upon confluency, cells were incubated with increasing concentrations of [^125^I]-mAb (3.9 pM–50 nM in complete medium) alone (“solo”) or in the presence of fixed concentration (25 nM) of unlabeled paired mAb(“paired”), in triplicates at 4°C for 2 h. At the end of incubation, cells were washed five times with ice-cold washing buffer (3% BSA in PBS). The cell-associated radioactivity was measured by a gamma counter (Wizard^2^ 2470, Perkin Elmer, MA), and was normalized to the total number of cells.

### RIA with live-cell cellular homogenates

Live-cell cellular homogenates were coated on 96-well Multiscreen Filter Plate, Immobilon P (EMD Millipore, Darmstadt, Germany) at a concentration of 0.67 mg/mL (100 μL each well) for 2 h at 37°C. Wells were triple washed with washing buffer (10% (v/v) FBS in PBS) using vacuum filtration with MultiScreen Vacuum Manifold (EMD Millipore, Darmstadt, Germany). To block non-specific binding, wells were incubated with blocking buffer (1% (w/v) BSA in PBS) for 2 h at 37°C with subsequent triple washes as described. Wells were then incubated with increasing concentrations of [^125^I]-mAb (1.6 pM–20 nM in assay buffer) “solo” or “paired” with fixed concentration of unlabeled mAb (25 nM) in triplicates at RT for 2 h at RT with mild shaking of the plate. To stop binding reaction, cells were filter-washed four times with ice-cold washing buffer. Non-specific binding (NSB) was calculated by measuring radiolabeled ligand binding to wells coated with 3% (w/v) BSA in PBS. Experimental data was measured by a gamma counter (Wizard^2^ 2470, Perkin Elmer, MA), analysis was performed as described for live-cell RIA.

### RIA with immobilized protein

Recombinant Mouse CD31/PECAM-1 Protein (extracellular domain of muPECAM-1 with predicted molecular mass of 65 kDa) was coated on 96-well Multiscreen Filter Plate, Immobilon P (EMD Millipore, Darmstadt, Germany) at a concentration of 5 μg/mL for 2 h at 37°C. Subsequent steps of washing, blocking as well as RIA for the assessment of binding parameters and data analysis were performed as described above for cellular homogenates using vacuum filtration with MultiScreen Vacuum Manifold (EMD Millipore, Darmstadt, Germany).

### Calculation of binding constants

Binding characteristics were analyzed using Prism 5.0 software (GraphPad), as described elsewhere[11]. Briefly, NSB was calculated by measuring radiolabeled ligand binding to wild-type cells (live-cell assay) or wells containing no antigen (homogenate assay). The equilibrium binding constant (K_d_) and the number of functional binding sites (B_max_) were obtained using non-linear regression analysis of a one-site binding hyperbola:

$$SpecificBound\left(\frac{mAb}{cell}\right)=TotalBinding-NSB=\frac{B_{max}\times \left[X\right]}{\left[X\right]+K_{d}}-NSB$$

where B_max_ is the maximum number of binding sites per cell at the asymptotic maximum; X is [mAb], and K_d_ is the apparent equilibrium dissociation constant. K_d_ and B_max_ values represent the mean ± SD of three or more independent experiments with each experiment performed in triplicate. Significant differences between means were determined using one-way ANOVA followed by post-hoc Bonferroni multiple comparison test, or unpaired student t-test, as appropriate. *P*<0.05 was considered statistically significant. All curve fitting and statistical analyses was conducted using Prism 5.0 software.

## Results

### Collaborative enhancement is independent of PECAM-1 homophilic interactions in the cell plasma membrane

Endothelial PECAM-1 exists both as monomers in apical domains and as homodimers in intercellular junctions. Targeted mutagenesis identified amino acids involved in the IgD1/D1 homophilic interactions between two PECAM-1 molecules including D11, D33, K50, D51 and K89. In particular, the K89A mutation abolishes homophilic binding [21]. To test whether homophilic PECAM-1 interactions are involved in collaborative enhancement, we have used REN cells expressing full-lengh (RmP) and mutant forms (RmP_K89A_) of PECAM-1.

**Fig 1:**
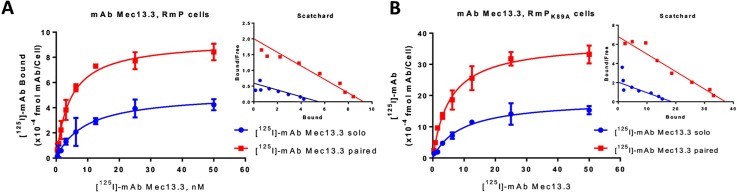
Collaborative enhancement binding of anti-muPECAM-1 [125I]-mAb Mec13.3 in live cells. Binding curves of [125I]-mAb Mec13.3 alone (“solo”) or in the presence of unlabeled mAb 390 to paired epitope (“paired”) in live cells stably expressing recombinant muPECAM-1 (RmP) (A) and mutant form of PECAM-1 (RmPK89A) (B) determined by RIA-based method. Increasing concentrations of [125I]-mAb treatments solo or with paired mAb were added to cells and incubated at 4°C for 2h. The results are presented as total binding corrected for nonspecific binding on REN-WT cells. Decrease in Kd corresponds to the increase in binding affinity. In RmP cells, Kd of Mec13.3 sees 0.48 ± 0.03-fold change in solo vs paired, P<0.001. In RmPK89A cells, Kd changed 0.06 ± 0.05-fold, P<0.001. The insets show Scatchard analysis of experiments.

**Fig 2:**
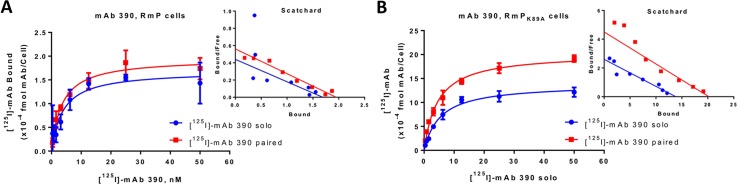
Collaborative enhancement binding of anti-muPECAM-1 [125I]-mAb 390 in live cells. Binding curves of anti- [125I]-mAb 390 alone (“solo”) or in the presence of unlabeled mAb Mec13.3 to paired epitope (”paired”) in live cells stably expressing recombinant muPECAM-1 (RmP) (A) and mutant form of muPECAM-1 (RmPK89A) (B) determined by RIA-based method. Increasing concentrations of [125I]-mAb treatments solo or with a paired mAb were added to cells and incubated at 4°C for 2h. The results are presented as total binding corrected for nonspecific binding on REN-WT cells. The effect of co-incubation with paired mAb vs solo was not significant. Yet, Kd changed 0.86 ±0.07-fold in RmP and 0.83±0.05 in RmPK89A. The insets show Scatchard analysis of experiments.

Previously we found that mAbs Mec13.3 and 390 bind to adjacent, but non-overlapping epitopes in IgD2 of mouse PECAM-1 with different affinities (IC_50_ = 2.5 nM vs 0.1 nM, respectively, according to analysis of binding curves of indirect ELISA) [11]. In the present study we assessed directly binding of [^125^I]-labeled mAbs ([^125^I]-mAbs). **Fig 1A and 1B**and **Fig 2A and 2B**show individual and paired binding curves of these mAbs to REN cells expressing wild type and K89A mutant forms of mouse PECAM-1, whereas **Table 1**shows quantitative parameters of K_d_ and B_max_. The decrease in absolute values of K_d_ of [^125^I]-mAb incubated solo or with paired mAb corresponds to the increase (enhancement) of apparent binding affinity.

**Table 1:** Collaborative enhancement binding of anti-muPECAM-1 [125I]-mAb in live cells stably expressing full-length (RmP) and mutant forms (RmP_K89A_) of muPECAM-1.

	B_max_, 10^−4^ fmol mAb/cell		K_d_, nM	
	RmP	RmP_K89A_	RmP	RmP_K89A_
Mec13.3 solo	5.2±0.4	18.7±1.1	8.8±0.7	9.1±1.1
Mec13.3 paired	9.3±0.3	37.3±1.3	4.2±0.3	5.5±0.4
CEPAL, fold change	1.79±0.09*	2.00±0.08*	0.48±0.03*	0.60±0.05*
390 solo	1.7±0.1	13.8±0.4	4.2±0.5	5.4±0.4
390 paired	1.9±0.1	20.3±0.5	3.6±0.2	4.5±0.3
CEPAL, fold change	1.12±0.05*	1.47±0.03*	0.86±0.07	0.83±0.05

Binding parameters, Kd and Bmax, of anti-muPECAM-1 mAbs Mec13.3 and 390 in live cells are presented as the mean ±SD of three or more independent experiments performed in triplicates. Collaborative enhancement effect (CEPAL) is presented as the fold change between [125I]-mAb binding with paired mAb, solo binding taken as a baseline. The CEPAL-induced changes in binding affinity Kd of (125I)-mAb was not mutual: in vitro, mAb 390 has an effect on binding of Mec13.3, but not vice versa. Interestignly, the amount of binding sites Bmax increased for all CEPAL conditions. Fold change is presented as a change of paired vs solo, ±SD (*, P<0.001).

Paired mAb 390 changed the K_d_ of [^125^I]-mAb Mec13.3 both in RmP cells by (0.48 ± 0.03)—fold (*P*<0.001) (K_d_ of solo vs paired binding 8.8 vs 4.2 nM, respectively) and in RmP_K89A_ cells by (0. 6±0.05)-fold (*P*<0.001) (K_d_ of solo vs paired binding 9.1 nM vs 5.5 nM, respectively). This phenomenon was not reciprocal; unlabeled mAb Mec13.3 had rather negligible effect on binding of [^125^I]-mAb 390 both in RmP cells (K_d_ 3.6 nM vs 4.2 nM for paired vs solo [^125^I]-mAb 390) and in RmP_K89A_ cells (K_d_ of solo vs paired binding 5.4 nM vs 4.5 nM, respectively). Despite the fact that there were no significant changes in binding affinities of [^125^I]-mAb 390 in the presence of mAb Mec13.3 vs solo, it was adjusted by (0.86±0.07)-fold in RmP and (0.83±0.05)-fold in RmP_K89A_ cells.

As expected, transgene expression levels are different in cells transfected with different genetic constructs. However, paired mAbs affected B_max_ of labeled counterparts in both types of cells. Binding of [^125^I]-mAb Mec13.3 in the presence of mAb 390 has shown a (1.79±0.09)- and (2.00±0.08)-fold increase in B_max_ values (*P*<0.001) in RmP and RmP_K89A_ cells, respectively. For [^125^I]-mAb 390 paired with mAb Mec13.3, this effective increase in B_max_ values was (1.12±0.05)-fold (in RmP cells) and (1.47±0.03)-fold (RmP_K89A_ cells) over solo binding (*P*<0.001) (**Table 1**).

These data argue against a major role of homophilic PECAM-1 interaction in mechanism of collaborative enhancement. In fact, pattern of solo vs paired binding to mAb 390 in RmP_K89A_ cells implies that the stimulation of binding is rather more pronounced in the cells expressing PECAM-1 mutant lacking of homophilic interactions.

### Anti-muPECAM-1 [^125^I]-mAb binding in cellular homogenates of live cells is enhanced by paired mAb

**Fig 3:**
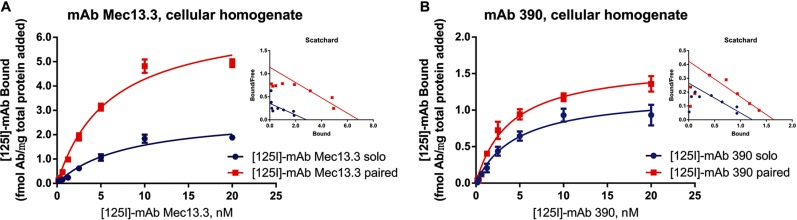
Anti-muPECAM-1 [125I]-mAb binding in cellular homogenates of live cells is enhanced by paired mAb. Binding parameters of anti-muPECAM-1 mAbs (A) Mec13.3 and (B) 390 in cellular homogenates of live cells stably expressing muPECAM. Membrane preparations were added to antibody cocktail [125I]-mAb solo or with paired mAb and incubated for 2h, followed by filtering through Millipore Multiscreen Filter Plates using vacuum manifold. The results are presented as total binding corrected for nonspecific binding on membrane preparations of REN-WT cells. Kd of Mec13.3 sees 0.77 ± 0.02-fold change in solo vs paired. Notably, Bmax values increase for both mAbs Mec13.3 and 390 co-incubated with a paired mAb. The insets show Scatchard analysis of experiments.

PECAM-1 plays an important role in endothelial signaling (8). In theory, mAbs can induce cellular activation bypassing interference in homophilic PECAM-1 interaction(s), by, for example, altering conformation, distribution in the cell membrane, or interaction with other cellular components. We tested whether collaborative enhancement involves cell signaling, by using a cell membrane homogenate preparation where cell signaling would be abolished. mAb binding studies were performed cell membrane vesicles obtained from REN mouse PECAM-1 cells (**S2 Fig**). In this model, [^125^I]-labeled mAb Mec13.3 and mAb 390 bind to membrane with K_d_ of 9.4 nM and 5.8 nM, respectively (corresponding B_max_ values are 2.3 fmol mAb/ μg total protein added and 1.25 fmol mAb/ μg total protein added) (**Fig 3A and 3B**).

Unlabeled mAbs enhanced binding of paired [^125^I]-mAbs in membrane vesicles like in intact cells: apparent binding affinity K_d_ changed (0.77±0.02)-fold for [^125^I]-mAb Mec13.3 co-incubated with mAb 390 (p<0.001), while mAb Mec13.3 provided no change in K_d_ of [^125^I]-mAb 390. Paired unlabeled mAbs increased the B_max_ (2.69±0.36)- and (1.30±0.02)-fold for [^125^I]-mAb Mec13.3 and [^125^I]-mAb 390, respectively (*P*<0.001) (**Table 2**).

**Table 2:** Collaborative enhancement binding of anti-muPECAM-1 [125I]-mAb in cellular homogenates of live cells stably expressing muPECAM-1.

	B_max_,fmol mAb/μg total protein added	K_d_, nM
Mec13.3 solo	2.3±0.5	9.4±0.2
Mec13.3 paired	6.2±1.0	7.2±0.2
CEPAL, fold change	2.69±0.36*	**0.77**±0.02*
390 solo	1.25±0.03	5.8±1.1
390 paired	1.62±0.02	5.7±1.9
CEPAL, fold change	1.30±0.02*	0.98±0.22

Binding parameters, Kd and Bmax, are presented as the mean ± SD of three or more independent experiments performed in triplicates. Fold change is presented as a change of paired vs solo, ±SD. (*, P<0.001).

### Collaborative enhancement in mAbs binding to recombinant PECAM-1

**Fig 4:**
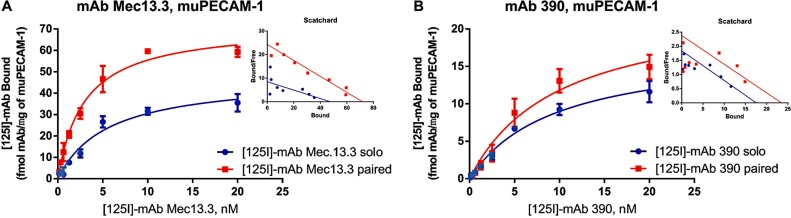
Anti-muPECAM-1 [125I]-mAb binding to purified muPECAM is enhanced by paired mAb. Binding parameters of anti-muPECAM-1 mAbs (A) Mec13.3 and (B) 390 with recombinant purified muPECAM-1. Millipore Multiscreen filter plates were coated with 5 μg/ml of recombinant muPECAM-1 and incubated for 2 h at 37°C. Radioligand binding assay was performed as described for cellular homogenates. The results are presented as total binding corrected for nonspecific binding on Filter Plates coated with 1% BSA. Kd of Mec13.3 sees 0.53 ± 0.03-fold change in solo vs paired. Again, Bmax values increase for both mAbs Mec13.3 and 390 co-incubated with a paired mAb. The insets show Scatchard analysis of experiments.

This data disproves a role that cell signaling plays in collaborative enhancement. Yet, the membrane preparation used in this study might contain residual machinery for cellular activation [22]. Further, mAbs binding to PECAM-1 anchored in this membrane preparation can alter its distribution or/and interaction with other membrane components. In order to ultimately exclude influence of any cellular components and of the membrane itself, we tested binding of mAbs to purified recombinant extracellular portion of PECAM-1, immobilized in the plastic. In this system, [^125^I]-mAb Mec13.3 and [^125^I]-mAb 390 had K_d_ of 5.3 nM and 9.2 nM, respectively. Maximal binding was close to 50 fmol and 16 fmol of mAb per μg of PECAM added (**Fig 4A and 4B**).

Unlabeled mAbs enhanced the binding of paired radiolabeled mAbs to purified PECAM-1. Similarly to binding in intact live cells, mAb 390 changed (0.53±0.03)-fold the K_d_ of [^125^I]-mAb Mec13.3 (p<0.001). Recapitulating behavior of reverse pairing observed in other models, enhancement was not mutual: mAb Mec13.3 had little, if any effect on binding of [^125^I]-mAb 390 (**Table 3**). At the same time, the number of binding sites B_max_ has increased nearly 1.5-fold for both sets of mAbs (p<0.001). This pattern is observed in all three experimental settings that agrees with the previously described hypothetical model of CEPAL where binding of a paired mAb causes conformational changes in PECAM making the second epitope more accessible.

**Table 3:** Collaborative enhancement binding of anti-muPECAM-1 [125I]-mAb in purified muPECAM.

	B_max_,fmol mAb/μg muPECAM-1 added	K_d_, nM
Mec13.3 solo	50.2±4.4	5.3±0.3
Mec13.3 and 390	75.0±4.5	2.8±0.2
CEPAL, fold change	1.5±0.09*	0.53±0.03*
390 solo	16.3±1.8	9.2±0.5
390 and Mec13.3	24.2±1.1	8.8±1.6
CEPAL, fold change	1.49±0.1*	0.96±0.11

Binding parameters, Kd and Bmax, are presented as the mean ± SD of three or more independent experiments performed in triplicates, for mAb Mec13.3 and mAb 390. Fold change is presented as a change of paired vs solo, ±SD. (*, P<0.001).

## Discussion

Unique features of both PECAM-1 structure and function have been revealed through use of monoclonal antibodies (24). In some cases, the structure/function relationships uncovered by mAb mapping have been relatively straightforward—for example, epitope mapping of a panel of antibodies which block neutrophil recruitment *in vivo* revealed the regions of the N-terminal Ig-like domain (IgD1) involved in leukocyte adhesion [23]. In other cases, mAbs have revealed more complex and unexpected relationships. For example, two antibodies (4G6 and 1.2) to the membrane-proximal IgD6 domain appear to enhance homophilic binding affinity of PECAM-1, despite the lack of direct involvement of IgD6 in the homophilic binding domain, the structure of which has recently been determined (Mei et al JBC 2014 and the crystal structure reference). Whether these antibodies induce a conformational change or affect its adhesive properties through other mechanisms remains unclear.

The recently described CEPAL effect may represent yet another level of complexity in probing the structure and function of cell adhesion molecules–in this case, through the use of paired mAb directed against adjacent, but distinct epitopes. For example, it was found that not all epitopes are displayed on PECAM-1 equally; the higher affinity of mAb was accompanied by lower epitope accessibility [11].

The complexity of the CEPAL phenomenon was recently demonstrated using polystyrene nanoparticles coated with mAbs Mec13.3 or 390 and co-administered in a endothelial HUVEC cell culture system [24]. The finding that fluid shear stress potentiates the CEPAL effect, suggested a potential role for PECAM-1 involvement mediated through mechanosensing and cell signaling. At the same time, CEPAL was not affected by the treatment with sodium azide/2-deoxyglucose, indicating that endocytosis or other energy-consuming processes are not involved. The effect was completely abolished, however, by fixation with paraformaldehyde, suggesting that a conformational change, such as the disruption of PECAM-1 homodimers, might be responsible for enhanced accessibility of paired antibody to its epitope.

The current report presents a further in-depth investigation of the CEPAL phenomenon. The CEPAL effect was preserved in homodimer-deficient K89A mutant form of PECAM-1, as well as in systems lacking cell signaling components, including cellular homogenates and recombinant PECAM-1; there was a consistent change in binding parameters throughout the panel of different experimental setups (**S3 Fig**). These experiments confirmed earlier *in vitro* studies, showing that the enhancement effect of paired mAb 390 is observed when introduced into systems probing mAb Mec13.3 binding, whereas there is no appreciable effect of mAb Mec13.3 on mAb 390 binding. Interestingly, *in vivo* studies in mice highlighted an opposite mutual effect in the tandem of these pairs of antibodies in previously reported experiments: the effects were more robust and unlike with *in vitro* studies, paired antibodies mutually exhibitied CEPAL, albeit Mec13.3 mAb was a stronger enhancer for CEPAL than 390 mAb [11]. Current findings support the hypothesis that the collaborative enhancement effect is the result of conformational changes in Ig-like protein structures. The binding of a paired ligand is the result of changes in IgD1 and IgD2 domains that makes the second epitope (hindered under normal conditions) more accessible. This feature is not likely to be exclusive to PECAM-1 molecule and/or endothelial cells.

Possible explanation of these changes is the formation of antibody-antigen complexes after binding of the first mAb that exposes the protein’s cryptic epitopes. Multiple experimental approaches, including crystallography, surface plasmon resonance, and isothermal titration calorimetry, have shown that the interaction of antibody molecules with specific epitopes on targeting proteins can lead to allosteric conformational changes in the antibody constant region [25,26]. At the same time, these interactions affect an antigen itself: if this antigen is of protein nature, then these newly formed antibody-antigen assemblies present an important class of protein-protein complexes derived from mutual conformational changes [27]. That way, these complexes present new antigens for binding of other ligands, including other antibodies, which makes it possible for us to observe their enhanced binding.

Cryptic epitope binding has also been associated with the behavior of other molecules. For example, the development of heparin-induced thrombocytopenia (HIT), where antibodies are only produced when a complex of heparin and platelet factor 4 (PF4) is formed. Interestingly, the role of cryptic epitope binding in a molecules’ behavior is not novel. Adjustments in protein antigen structure and exposure of partly occult epitopes is observed in binding of broadly neutralizing monoclonal antibodies (4E10 and 2F5) to the conserved membrane proximal external region (MPER), adjacent to the transmembrane domain of human immunodeficiency virus type-1 (HIV-1) [28], an attractive feature to be utilized in the production of anti-HIV vaccines. Cryptic epitope exposure plays a critical role in the development of reactivity of T- and B-cells against self-molecules. For example, hindered epitope binding is one of the mechanisms which allow an immune response to be directed against self-antigens [29]. In endothelial cells, blocking antibody (E4G10) recognizes VE-cadherin epitopes only accessible in neovasculature. It inhibits VE-cadherin function during tumor angiogenesis but does not disrupt existing adherens junctions on normal vasculature [30].

Perhaps most importantly, improved understanding of the mechanistic aspects of CEPAL will provide further understanding of the structure and function of PECAM-1 in vivo, which may in turn have implications for vascular immunotargeting. PECAM-1, has proven a robust surface target capable of delivery of diverse therapeutic molecules and genetic materials [8,31,32]. These practical applications fall into several categories, some of which have already been explored. For example, Chacko et al [24] investigated the most obvious application of CEPAL–enhanced delivery—demonstrating a 5.4-fold increase in targeting of PECAM-1 nanoparticles following pre-injection of paired antibody. Future studies are planned to determine if this effect can be utilized to increase delivery of therapeutic cargo and improve outcome in vascular disease. A second class of applications involves delivery of paired cargoes First demonstration of a positive modulatory effect of endothelial binding by using CEPAL effect were previously reported in the experiments involving therapeutic thrombomodulin fusion proteins with PECAM-1 targeted single chain fragment variable domains (7). Another example of endothelial directed tandem drugs that could have beneficial implications from CEPAL effect include antioxidant drugs such as SOD and Catalase[33,34].

## Conclusion

Collaborative enhancement effect was preserved in various systems including cellular homogenates and recombinant, purified muPECAM-1, providing evidence that it is independent of cell signaling. Moreover, collaborative enhancement binding was preserved in REN cells stably expressing mutant form of muPECAM-1 lacking homophilic binding. These new findings elucidate possible mechanisms underlying collaborative enhancement binding of Abs to PECAM-1, and suggest that this phenomenon may be observed in other members of Ig superfamily of cell adhesion molecules. Further studies to determine crystal structures and conformational changes in PECAM molecule during binding of paired mAbs will allow for the development of a detailed model to understand these changes on atomic level.

## Supporting Information

S1 Fig Characterization of REN cells expressing mutant PECAM-1 by fluorescence-activated cell-sorting (FACS). **(**A) REN cells transduced with wild type mouse PECAM-1 (RmP) were stained with FITC-labeled anti-PECAM-1 mAb Mec13.3. Isotype control is shown in dotted red. (B) REN cells transduced with REN-MP_K89A_ were stained with FITC-labeled anti-PECAM-1 mAb Mec13.3. Isotype control is shown in dotted red.
(EPS)

S2 Fig Membrane preparation of live cells: experimental design. (A) The PECAM-1 presence in cellular membrane homogenates is confirmed by western blot analysis. Anti-pan-cadherin mAb is used as a reference to cell membrane. (B) Step 1. Cellular homogenates of live cells are mixed with [^125^I]-mAb solo or with paired mAb. The binding assay is conducted in complete cell culture medium for 2hr at RT. Step 2. To terminate the binding reaction, the mixture was filter-washed four times with ice-cold washing buffer (PBS with 0.1% BSA), followed by immediate filtration using on Multiscreen Filter Plate, Immobilon P (EMD Millipore, Darmstadt, Germany) vacuum manifold (shown in green). Step 3. Radioactivity associated with the filters was quantified by gamma counting.
(EPS)

S3 Fig Summary for binding parameters (K_d_/B_max_) presented for all experimental setups. (A) K_d_ is presented in absolute values whereas (B) B_max_ values are normalized to the B_max_ of [^125^I]-mAb solo in each individual system (baseline level indicated in dash line). K_d_ is presented as the mean ± SD of three or more independent experiments performed in triplicates (*, *P*<0.001, n = 3).
(EPS)
